# Scripps Genome ADVISER: Annotation and Distributed Variant Interpretation SERver

**DOI:** 10.1371/journal.pone.0116815

**Published:** 2015-02-23

**Authors:** Phillip H. Pham, William J. Shipman, Galina A. Erikson, Nicholas J. Schork, Ali Torkamani

**Affiliations:** 1 Scripps Health, La Jolla, CA 92037, United States of America; 2 The Scripps Translational Science Institute, La Jolla, CA 92037, United States of America; 3 The Department of Integrative Structural and Computational Biology, The Scripps Research Institute, La Jolla, CA 92037, United States of America; 4 The Department of Molecular and Experimental Medicine, The Scripps Research Institute, La Jolla, CA 92037, United States of America; 5 Cypher Genomics, Inc., La Jolla, CA 92037, United States of America; West Virginia University, UNITED STATES

## Abstract

Interpretation of human genomes is a major challenge. We present the Scripps Genome ADVISER (SG-ADVISER) suite, which aims to fill the gap between data generation and genome interpretation by performing holistic, in-depth, annotations and functional predictions on all variant types and effects. The SG-ADVISER suite includes a de-identification tool, a variant annotation web-server, and a user interface for inheritance and annotation-based filtration. SG-ADVISER allows users with no bioinformatics expertise to manipulate large volumes of variant data with ease – without the need to download large reference databases, install software, or use a command line interface. SG-ADVISER is freely available at genomics.scripps.edu/ADVISER.

## Introduction

The availability of high-throughput DNA sequencing technologies has enabled nearly comprehensive investigations into the number and types of sequence variants possessed by individuals in different populations. For example, not only is it now possible to sequence a large number of genes in hundreds if not thousands of people, but it is also possible to sequence entire individual human genomes in the pursuit of inherited disease-causing variants or somatic cancer-causing variants [1–5]. The day where whole genome sequencing is a relatively routine procedure lies within the near future, as high-throughput sequencing costs and efficiency continue to improve at a blistering pace.

One particularly vexing problem that has accompanied the development and application of high-throughput sequencing is making sense of the millions of variants identified per genome. For example, recent successes at identifying variants associated with rare disease have generally required large bioinformatics teams—restricting the effective implementation of whole genome sequence-based clinical and research endeavors to large institutions and/or genome centers [2,3]. Similarly, while the GWAS strategy could potentially identify tag-SNPs explaining up to half the heritability of common diseases [6,7], sequence-based methods will likely be necessary for the identification of rare variants predisposing to common diseases where variable penetrance, allelic and locus heterogeneity, epistasis, gene-gene interactions, and regulatory variation play a more important yet elusive role. The sensitivity of set-based rare variant analyses to the inclusion of non-causal and exclusion of causal variants indicates a clear role for automated set generation and variant prioritization in these analyses [8,9]. Finally, the recent unveiling of the role of ultra-rare and/or *de novo* variants in the etiology of human disease, especially in idiopathic disease and neuropsychiatric disorders—or the vast number of somatic mutations that can perturb tumor suppressor function in cancer—suggests that reliance upon variants statistically associated with disease for molecular diagnosis at an individual level will be suboptimal in many instances [10–12]. The issue of interpretation of variants of unknown significance can only be expected to worsen as humans continue to postpone reproduction to more advanced age and the number of recently derived deleterious variants continues to explode [13]. Analysis of rare variants in these various scenarios is potentially addressable through holistic and accurate variant annotation.

A clear need for functional annotation has been recognized since investigators began searching for causal variants linked to GWAS tag-SNPs. Early tools developed for this purpose, built under the assumption that common variants would explain disease predisposition, are limited to databases which provide only information on known SNPs [14–17]. Novel and/or rare, *de novo*, and indel variants, are not accessible within this framework. More recently developed tools sensitive to the importance of undiscovered or more complex variants simply annotate variants based on the known genomic elements they reside and/or restrict functional predictions to pre-computed nonsynonymous variant functional predictions [18–24]. While these tools are immensely useful in their own right, none are capable of producing *predictions* for the near infinite possible variants generated in a sequencing project. We would like to emphasize the distinction between algorithmic prediction rather than simple determination of residence within genomic elements. For example, while missense SNP impact predictions via e.g., Polyphen [25] or SIFT [26], can be precomputed with relative ease [27], it technically impossible to precalculate the algorithmically predicted impact of all possible inframe indels on protein function or on transcription factor binding sites. A powerful webserver interface is required to enable this sort of *de novo* calculation. There is a clear need for a more holistic and integrated annotation tool to both annotate and predict the functional effects of the numerous variant classes produced by whole-genome sequencing projects and allow for the processing of those predictions alongside genotype data. The tool presented here, Scripps Genome ADVISER, aims to fill this role in a manner accessible to research endeavors at all levels of bioinformatics sophistication.

## Methods

### Overview

**Fig 1:**
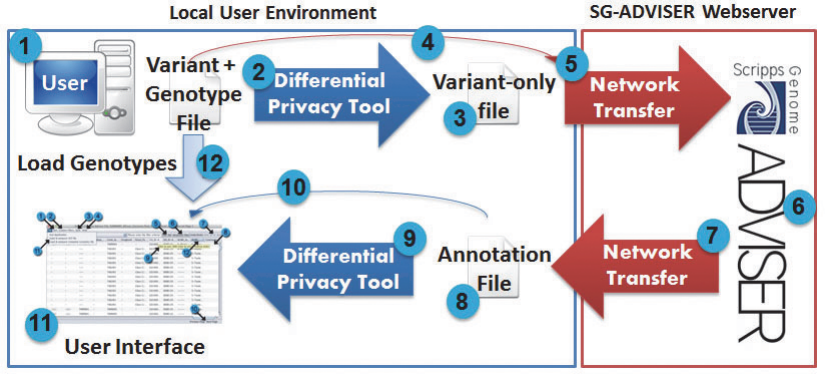
SG-ADVISER Suite and Workflow. This figure depicts the workflow for variant annotation and analysis. Beginning with a user with a file containing variant and genotype information (1), the user can optionally use the privacy tool (2) to generate a variant-only file with (3) with genotypes removed and clinically relevant variants implanted. This file, or the original variant file if desired, is then uploaded to the SG-ADVISER webserver (4,5). The SG-ADVISER webserver performs variant validation and annotation (6). If the file contains errors, the validated variants may be automatically resubmitted to the webserver. At the completion of annotation, annotation information is downloaded from the webserver down to the users local environment (7). The resultant annotation file (8) can then be run in reverse through the privacy tool (9) to remove implanted clinical relevant variants. The resultant file or the original annotation file is then loaded into the user interface (10,11). Finally and optionally, genotype information from the original variant file can be loaded into the user interface (12). The genomic data is ready for downstream analysis.

SG-ADVISER is a multi-component system (Fig. 1) including: 1) a privacy tool for markedly reducing or eliminating the usefulness of genomic data should it be intercepted in transit to the webserver, 2) a webserver that accepts and returns genomic data and annotations, 3) a variant validation and correction system that accepts and converts various variant file formats, and validates and/or corrects the accuracy of variant information against the reference genome with informative error and corrections reporting and the option to immediately resubmit valid and corrected variants, 4) a high-performance computing system that utilizes both pre-computation databases and parallel computations to produce variant annotations rapidly, and 5) a local client graphical user interface that allows loading of genotype information and the filtration of variants based upon annotations and comparisons of multiple genomes using custom as well as predefined variant filtration strategies. The overall goal is to provide near comprehensive variant annotation without the burden of complex software or intense client-side compute capabilities, while simultaneously maintaining the privacy of user data and avoiding over-simplification of the annotations themselves.

### Computational Infrastructure

Annotation proceeds in highly parallel fashion and includes classes of variant annotations that are entirely independent of one another, serially dependent annotations whose execution are dependent upon the completion and status of prior annotations, and synthetic annotations that generate new information through the combination of multiple annotation outputs. In contrast to existing tools which rely upon independent lookup tables, SG-ADVISER can produce a virtually infinite range of different annotation outputs depending upon the nature of the submitted variants. These processes are computed de-novo for any variants not previously observed in any genome, while annotations for previously observed variants are retrieved from a pre-annotation database. De-novo annotations are executed on a cluster of five Dell PowerEdge servers configured with 16 cores and eight terabytes of local disk space per server. Once completed, the new annotations are stored in the pre-annotation database for fast lookup of subsequent occurrences. The pre-annotation database is stored in MongoDB a NoSQL format database divided into separate collections by chromosome and indexed by 5 variant characteristics [start coordinate, end coordinate, variant type, reference allele, alternate allele]. The pre-annotation database currently contains over 220 million variants, consisting mostly of variants observed in the 1,000 Genomes Project, NHLBI exome sequencing project, dbSNP, and the Scripps Wellderly cohort [28–30]. For a detailed description of the computational processes underlying SG-ADVISER see *S1 Text*.

### Performance

The computational infrastructure underlying SG-ADVISER allows rapid turn-over of a single whole genome variant files. We evaluated the performance of SG-ADVISER by annotating 10 whole Wellderly genomes, sequenced by Complete Genomics, and not previously annotated by the SG-ADVISER system. At an average of 4,091,804 unfiltered variants per genome, the average time to completion was 110 ± 9 minutes. Exomes complete in considerably less time, at an average of 112,008 unfiltered variants per exome (10 exomes total), variant annotation completed in 24 ± 6 minutes per exome. One caveat to this performance is that only one variant file at a time can be processed, occupying the entire computational cluster, thus real turn around times can be dependent upon user traffic. However, as the number of variants annotated by SG-ADVISER increases, performance and turn-around-time is expected to improve further.

### Data Input Formats

SG-ADVISER supports human genome annotation (hg19) only. SG-ADVISER accepts variant files in VCF, Complete Genomics, or plain tab-delimited file formats. For most accurate results variants should be submitted in 0-based coordinates with positive strand nucleotides reported. However, given our experience with the numerous variant input formats provided by early users, the SG-ADVISER validator will attempt to determine whether variants are 0-based or 1-based by evaluating matches to the reference genome, and convert coordinates appropriately. Moreover, the SG-ADVISER validator will attempt to correct reference-alternate allele swaps and/or nucleotides reported relative to the negative strand. While the presence of any incorrectly formatted variants will stop the automated annotation process—a descriptive error file is produced with the option of automatically resubmitting the corrected variants. The nature of the applied correction is provided in the final annotation output. Often times errors can be produced due to conflicting reference genome coordinates—rather than annotating this variants regardless of the reference match, an error is produced but annotation can be continued on all other verified variants. For more information on input formats and error reporting, see: http://genomics.scripps.edu/ADVISER/Input_Desc.jsp.

### Data Output Format

Annotations are output in a tab separated file, where the first eight columns contain information about the submitted variant itself, and the rest of the columns are annotations produced by SG-ADVISER. Variants are presented as a single line per variant, yet complete annotations are produced for each individual transcript influenced by a variant, thus the format of each annotation column depends upon whether the annotation is relative to the gene or transcript it impacts or relative to the physical location of the variant. Any column containing annotations produced relative to a gene or transcripts are further subdivided by triple back slashes ("///"). Across annotation columns, “///” separated values correspond to one another—i.e. annotations in the same position relative to “///” separated values within a column influence the same transcript. Annotations not directly relevant to a particular transcript, for example transcription factor binding sites or the conservation of the position, are also “///” separated but that separation corresponds to a related column. For example, transcription factor binding sites influenced by a variant are “///” separated, and the calculation of the impact of the variant on binding of the “///” factor is presented in a separate “///” separated column. When an annotation is not applicable to a variant or transcript, a null value is represented by a "-" character, often in the format of the column. For example, a column where entries are formatted as "Value1∼Value2", if null, will receive a value of "-∼-". This is required due to partially complete outputs, for example where only one of two output values is null. For a more thorough description of the annotation types and output format, see *S1 Text* and http://genomics.scripps.edu/ADVISER/Result_Desc.jsp

### Security and Privacy

Data is encrypted during transfer to SG-ADVISER via a Secure Socket Layer (SSL 3.0) to a secure computational cluster maintained by The Scripps Research Institute. Thus, SG-ADVISER is compliant with the dbGaP Security Best Practices for controlled access data. Additionally, variant files uploaded to SG-ADVISER, as well as the resultant annotation file, are destroyed 30-days after variant file upload. To ensure confidentiality of valuable research data, we do not retain any information about the number, identity, or combinations of variants submitted by any user. As mentioned previously, annotations for each individual variant are stored in a pre-computed annotation database to improve the speed of future annotation, but no information beyond the physical location of the variant is retained—no association between variants in the pre-annotation database and the source or additional observations of the variant is preserved.

To facilitate and improve privacy further, a privacy tool is available for download at (http://genomics.scripps.edu/ADVISER/PrivacyTool.jsp). This tool will automatically strip genotype information from VCF files for users without the bioinformatics means to do so. Genotype information is not required for SG-ADVISER annotations—thus, removal of genotype information from uploaded files is suggested for sensitive genomes. However, because we suspect it is nearly impossible to de-identity a genome without information loss we have designed our privacy tool render any transit or server-side data interceptions uninformative [31]. The SG-ADVISER privacy tool will implant known clinically relevant variants into a variant file processed by the tool—making the identification of true vs. implanted clinically informative variants impossible. These variants can then be removed from the annotation file on the client side through the privacy tool by referencing the original VCF file. Thus, overall control of privacy remains in the hands of the end user with the original variant file, which need not ever be transferred to the SG-ADVISER web-server.

## Results

### Annotation Categories

At its core, SG-ADVISER is an automated computational system for producing known and predicted information about genetic variants—otherwise known as variant annotations. SG-ADVISER produces four major classes of variant annotations including: 1) residence within known or inferred genomic elements (e.g., exons, promoters, conserved elements, transcription factor binding sites, protein domains etc.); 2) annotation and prediction of the functional impact of a variant on genomic elements (prediction of impact on protein function, changes in transcription factor binding strength, splicing efficiency, microRNA binding, etc.); 3) annotation of molecular and biological processes which link variants across genes and/or genomic elements with one another, and 4) annotation of known or predicted population-based, clinical, and/or molecular characteristics of the gene or variant (e.g. population frequency, pharmacogenetic variants, disease associations, eQTLs etc.). Detailed descriptions of the 70+ specific annotations are provided in *S1 Text* and are available at (http://genomics.scripps.edu/ADVISER/Result_Desc.jsp). Key highlights include:

1. SG-ADVISER produces predictions for the functional impact of numerous variant types including; nonsynonymous variants, in-frame variants, truncating variants, splice site variants, microRNA binding site variants, transcription factor binding site variants, and the changes in microRNA targets induced by variants within microRNAs themselves. As previously emphasized—these annotations are not limited to classification as one of the above types of variants or residence within a motif or pre-defined site, but rather classification plus a *prediction* as to whether the variant functionally impacts the genomic element they resides in.
2. Allele frequency information from the 1000 Genomes Project [28], NHLBI Exomes Project [29], and the Scripps Translational Science Institute Wellderly cohort are disseminated through SG-ADVISER. The Wellderly cohort is composed of individuals over the age of 80 with no common chronic conditions. 400+ individuals have been whole genome sequenced by Complete Genomics. Their allele frequencies are available through SG-ADVISER and will continue to be updated as the cohort continues to be sequenced.
3. Prior knowledge from the Human Gene Mutation Database (HGMD) [32], OMIM [33], Clinvar [34], the Genetic Association Database and GWAS Catalog [35,36], and the Catalogue of Somatic Mutations in Cancer [37] are provided. HGMD license information is required for the return of results from HGMD.
4. A synthesis of the above produces an American College of Medical Genetics-like (ACMG) ADVISER variant classification schema for known and predicted disease associated.

### ADVISER Variant Classification

Two different modified American College of Medical Genetics (ACMG) variant classifications are produced, one based upon variants, or variants in genes known to be causally associated with a phenotype (ADVISER Clinical) and a second score which includes genes known to carry genetic variants that are statistically associated risk factors for the development of a disease (ADVISER Research). The ACMG scoring guidelines, with categories 1–6, are modified and expanded to include a 1*, 2* and 4* category to provide more granularity to variant stratification, for example by down weighting reported pathogenic variants to category 1* based on allele frequency, or by allowing for stratification of variants of the same functional class (e.g. missense variants) across the ADVISER classes based on algorithmic predictions of pathogenicity rather than relegating all nonsynonymous variants unreported as pathogenic to variants of unknown significance [38]. Variants of category 1–2* are of most clinical relevance and category 6 contains common risk factors for disease. The details for ADVISER classes are defined in *S1 Text* and will be updated at (http://genomics.scripps.edu/ADVISER/ACMG.jsp). In brief, ADVISER category 1 variants are rare (<1% allele frequency) reported pathogenic variants. Category 1* includes more common (1–5% allele frequency) reported pathogenic variants—which tend to be either false positive reports or variants with incomplete penetrance or acting as modifiers. Category 2 contains rare variants in known disease genes, unreported as pathogenic, but predicted to impact gene function by either removing a splice site donor or acceptor, producing an amino acid substitution predicted to functionally impact the protein, or truncating the protein in a damaging manner. Category 2* includes rare truncating variants not predicted to damage protein function or uncommon truncating variants predicted to damage protein function. Allele frequencies are determined using the maximum allele frequency across our previously described reference populations.

The performance of the ADVISER classification schema was evaluated by categorizing a set of known high confidence nonsynonymous disease causative and neutral polymorphisms derived from the SWISS-PROT feature table [39]. 16,549 variants classified as disease causative (positive class) and 11,282 variants classified as neutral polymorphisms (negative class) in known disease causative genes were compiled in order to determine how well the SG-ADVISER classifications recapitulated the SWISS-PROT classifications at various SG-ADVISER class thresholds. Variants are considered true positive if a SWISS-PROT disease causative variant achieves a threshold ADVISER class or better (as delineated in Table 1). True negative variants are SWISS-PROT neutral polymorphisms not achieving the threshold ADVISER class or better. For example, a variant classified as disease causative in SWISS-PROT and achieving an ADVISER class of 2 would be considered a false negative at the ADVISER class 1 threshold, true positive at the ADVISER class 1–2 threshold, and true positive at the ADVISER class 1–3 threshold. None of the previously described mentioned annotation tools [18–24] produce overall variant categorizations, therefore, performance was compared to a popular commercial platform for variant analysis, Ingenuity Variant Analysis, under its default settings. As can be seen in Table 1, the SG-ADVISER schema provides a superior and more useful way of capturing potential disease associated variants in a manner that is tuned to relevant use cases. That is, while SG-ADVISER’s overall balanced accuracy (mean of sensitivity and specificity) is significantly but not dramatically superior, the specificity-sensitivity profile fulfills the actual requirements for practical use cases with dramatically superior specificity for the high confidence pathogenic categories and much more sensitive results for the lower confidence categories. In other words, when producing known or expected disease causative mutations (ADVISER class 1 and 2), SG-ADVISER’s superior specificity reduces false positive disease associations in a context where false positive results are unacceptable—for example when performing predictive molecular diagnosis in the absence of a disease phenotype. Similarly, in a less conservative scenario (ADVISER class 3), SG-ADVISER’s accuracy profile is more heavily weighted towards sensitivity, or inclusiveness of potential disease causative variants without unduly introducing false positive results—dramatically boosting negative predictive value. This accuracy profile is more useful in the case where a molecular diagnosis is to be made for an already present phenotype. Overall, the sensitivity-specificity profile of SG-ADVISER summary determinations are superior and address end-user needs in a more meaningful way by transitioning appropriately from conservative, high confidence, disease associations to comprehensive, high coverage, variant reports while maintaining superior accuracy overall.

**Table 1:** ADVISER Class Performance.

SG-ADVISER			Ingenuity Variant Analysis		
ADVISER Class	Sensitivity	Specificity	Metrics	Sensitivity	Specificity
1	83%	95%	Clinical Assessment	91%	83%
1–2	94%	86%	Clinical Assessment or Damaging by SIFT and Polyphen	92%	80%
1–3	98%	72%	Clinical Assessment or Damaging by SIFT or Polyphen	94%	72%

SG-ADVISER Classification performance. The Ingenuity Clinical Assessment considers variants classified as Known Pathogenic or Likely Pathogenic by Ingenuity. The default Ingenuity allele frequency threshold of 3% plus damaging predictions by SIFT and/or Polyphen were used to simulate less confident variant classification tranches in Ingenuity.

### Comparison to Other Methods

The ADVISER class performance evaluation described above considers only nonsynonymous variants, yet, the accuracy and comprehensiveness of SG-ADVISER annotations extend beyond to other important variant classes (Table 2). Truncating variants (nonsense or frameshift) are not evaluated any further by all available tools, yet it is known that the proximal and distal ends of genes are enriched in presumably neutral truncating variants [40]. Therefore, an algorithmic method to prioritize truncating variants based on the percentage of the conserved portion of the protein removed by the truncating variant after adjustment for alternative start sites, is incorporated in SG-ADVISER [41]. Similarly, in-frame indels are often considered neutral or not stratified in anyway by other tools, yet important disease causative in-frame indels, such as F508del-CFTR—the most common cause of cystic fibrosis—are well established. SG-ADVISER annotations algorithmically prioritize inframe variants [42]. This approach is amenable to, and will be extended to, the annotation of phased combinations of variants as phased genomes gain in prominence [43]. Finally, approximately 40% of known disease causative variants in HGMD that influence splicing do not impact the conserved splice-donor and acceptor nucleotides—yet, there is no way to prioritize variants nearby intron-exon junctions in available annotation tools. SG-ADVISER annotations prioritize these variants appropriately [44]. These differences extend beyond coding variants to the prediction of changes in transcription factor binding site affinity, via calculation of the change in score for a mutated sequence using position-specific scoring matrices, microRNA binding strength, and altered targets due to variants in microRNAs themselves via recalculation of targets, albeit at lower confidence than the above described predictions. No previously described methods offer these predictions.

**Table 2:** Annotation Tool Comparison.

Tool	Local Install vs. Web Service	Encryption (web)	Graphical User Interface	Variant Filtration	Summary Pathogenicity Score	Truncating variant effect prediction	TFBS variant effect prediction	In-frame variant effect prediction	Splice-site variant effect prediction	microRNA variant effect prediction	Custom Annotation
ANNOVAR	Both	No	No	Yes	Reported only	No	No	No	No	No	Yes
AnnTools	Local	N/A	No	No	Reported only	No	No	No	No	No	Yes
VEP	Both	No	Yes	Yes	Reported only	No	Yes	No	No	No	Yes
SeattleSeq	Both	No	Yes	Yes	Reported only	No	No	No	No	No	No
SeqAnt	Web	No	Yes	Yes	No	No	No	No	No	No	No
SVA	Local	N/A	Yes	Yes	Reported only	No	No	No	No	No	Yes
SG-ADVISER	Web	Yes	Yes	Yes	Reported and Predicted	Yes	Yes	Yes	Yes	Yes	No
SnpEff	Local	N/A	No	Yes	Reported only	Yes	No	No	No	No	Yes
VARIANT	Web	No	Yes	Yes	Reported only	No	No	No	No	No	No

Prediction refers to a determination of functional effect of a specific variant type—not simply whether the variant belongs to that type. Splice-site variant effect prediction refers only to non-donor/acceptor nucleotide predictions.

A number of tools described as variant annotation tools exist. Table 2 provides a comparison of SG-ADVISER functionalities with similar tools [45–47]. These tools generally predict variant effect by simply identifying overlap with pre-defined bins. Where tools, such as VEP, SnpEff, and ANNOVAR [18–20] incorporate algorithmic predictions, they do so through the inclusion of precalculation tables—thus practically limiting annotations to what can be precalculated (for example SIFT and Polyphen predictions), but allowing for more efficient expansion to other organisms. Similarly other tools, such as BEDTools [21], TREAT [22], SeqAnt [48], and AnnTools [23] simply allow for the overlap of variant coordinates with reference genes or intervals. Simple filters can be executed against the resultant annotations, but again, these tools rely upon the download of large pre-annotation databases and cannot be extended to more complex scenarios. Finally, tools such as GEMINI [49], Annotate-it [50], and VAR-MD [51] provide capabilities for more complex filtration strategies utilizing the basic annotations described previously. SG-ADVISER combines basic annotations, more complex annotations that require on the fly calculation, and complex filtration strategies enabled through the user interface.

### User Interface

**Fig 2:**
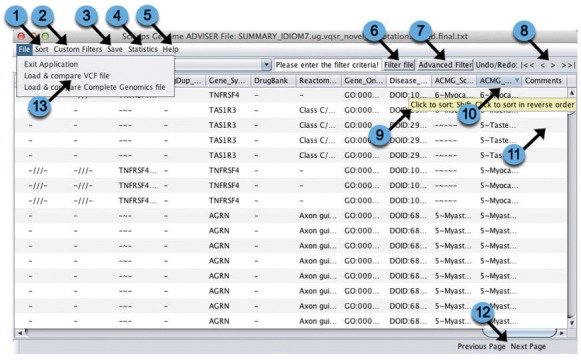
User Interface. The SG-ADVISER user interface provides a number of useful functionalities including: (1) sort the current view by any column; (2) 14 pre-defined custom filters, for example extraction of rare coding variants—for a list of custom filters see http://genomics.scripps.edu/ADVISER/downloads.jsp; (3) post-filtered files can be saved for manipulation outside of the UI; (4) calculation of variant type counts and frequency; (5) a help menu; (6) simple user-defined filter on a single column; (7) advanced multi-column user defined filtering; (8) the capability to move forward and backward through executed filters; (9) extensive tool tips; (10) sorting by clicking the column header; (11) the capability to add and save comments; (12) scrolling through the multiple pages of variants (1000 variants per page); (13) the ability to load in genotype data from the original variant file.

The SG-ADVISER user interface allows the user to load in an annotation results file, load in the genotypes for the annotated variants from the file submitted to the SG-ADVISER webserver (or from the original variant file passed through the SG-ADVISER privacy tool), and apply a wide variety of custom and pre-defined filters. The user interface is available at (http://genomics.scripps.edu/ADVISER/downloads.jsp)—and is built in Java to support cross operating system use. An annotated screenshot is displayed in Fig. 2. The user interface functionalities include: 1) basic sorting on any column, 2) basic filtration on any column, 3) advanced filters allowing specification of multiple columns linked by AND/OR operators, 4) capability to undo and redo actions, 5) application of custom pre-defined filters including inheritance based filters for family-based studies, 6) export of filtered files to be manipulated further by external tools, and 7) the calculation of summary statistics providing the number and rate of a wide variety of variant classes before or after the application of filters. The UI can load and process queries against a genome nearly in real-time: loading of exome data variant annotations for a trio, total of ∼145,000 variants takes ∼2 seconds, loading of the genotype data from a VCF file to be manipulated alongside annotations takes ∼4 seconds, and the execution of filters completes in less than 5 seconds for even the most complex queries. A standalone user interface has a few benefits: 1) whole genome variant filtration is impractical within a webserver, 2) on-the-fly computations such as variant summary statistics can be performed after the execution of customized filters, 3) genotype information can remain in the clients possession, and 4) variant filtration can be executed and saved for later processing. For a more detailed description of SG-ADVISER UI functionality, see *S1 Text*.

We believe the combination of holistic annotations and predictions provided by SG-ADVISER, plus the power to utilize those annotations alongside genotype information in the SG-ADVISER UI provides a powerful tool for the up-to-date processing of whole genome sequence information by individuals with little to no computational experience.

## Discussion

To our knowledge, SG-ADVISER is the most comprehensive and accurate annotation and variant filtration tool available. The overall goal of the SG-ADVISER suite of tools is to put computational power and bioinformatics expertise into the hands of individuals with little to no computational proficiency, but with the biological and/or clinical expertise to interpret genetic results when appropriately filtered, while protecting the privacy of study subjects. The annotations and filtration strategies enabled by the SG-ADVISER suite have been successfully used in the molecular genetic diagnosis of numerous idiopathic disease cases at The Scripps Translational Science Institute [52]. We hope to enable these sorts of investigations outside of the major genomics centers.

Furthermore, it is clear that sequence-based investigation into common disease will require the ability to accurately parse and prioritize regulatory variants. Therefore, we have placed some emphasis on building tools to not only determine whether a TFBS or miRNA binding site contains a variant, but whether that variant changes the function of that binding site in any meaningful way. Given the known sensitivity of set-based rare variant analysis methods to the inclusion of non-causal variants indicates, it is clear that automated set generation will require variant prioritization in order to achieve maximal power [8,9].

SG-ADVISER will continue be updated and expanded to provide access to new annotations/predictions as necessary. Questions and requests for specific annotations can be made on the Biostar forum http://www.biostars.org/.

## Supporting Information

S1 Text Detailed information about annotation types, annotation processes, and user interface functionality are provided. (DOC)
