# Supplementary material for: Scripps Genome ADVISER: Annotation and Distributed Variant Interpretation SERver
Source: PLoS One. 2015 Feb 23;10(2):e0116815. doi: 10.1371/journal.pone.0116815 (PMC4338027; doi:10.1371/journal.pone.0116815)
Supplement: S1 Text — (DOC) [file pone.0116815.s001.doc]

**Table of Contents**

**Annotation Classes………………………………………………………………………………1**

**Computational Processes..………………………………………………………………………5**

**User Interface………………………………………………………………………………….....8**

**References…..…………………………………………………………………………………...14**

**Annotation Classes**

**Physical and Gene-Relative Mapping and Characteristics**

The physical mapping information provides the most basic level of information regarding the location of the variant and its relationship with basic elements. The chromosome, physical start position, physical end position, variant type (e.g. snp, insertion, deletion, substitution) and reference and alternate alleles are supplied by one of a few standard file formats, including VCF (variant call format) (see http://www.1000genomes.org), Complete Genomics native file format, or basic tab delimited BED-like file format. Additionally, haplotype information, used to track whether variants occur in *cis* or *trans* relative to one another, can be provided and that information will be conserved. Subsequent instances of SG-ADVISER will take into account multiple variants on the same haplotype – however this information is not utilized currently. The above location information is utilized to execute a basic mapping step which determines what the nearest gene/transcripts are, what type of gene (coding vs. non-coding) is nearby, the location of the variant relative to the gene (exonic, intronic, upstream, downstream) and the distance from the gene-body. All determinations are based upon physical distances within the genome. Either known or predicted genes could be utilized for this step, in our specific implementation the UCSC Known Genes database is used (Fujita et al 2011) which is a compilation of information from RefSeq, GenBank, Consensus CDS project, UniProt, and other sources of evidence for genes and whether they are coding vs. non-coding genes.

**Coding Gene Impact, Inferred and Predicted**

If a variant is mapped to an exon of a protein coding gene, its impact upon the coding sequence is inferred based upon the standard rules of the genetic code, and prediction of impact is performed based upon a series of functional impact prediction algorithms. First, the position of the variant within the protein sequence and the distance of the variant relative to the N-terminal and C-terminal ends of the protein are determined – used in determining the impact of truncating variants. Next, the basic coding impact, e.g. synonymous, nonsynonymous, frameshift, etc. is determined as well as the original and variant amino acids based on the standard genetic code. Then, dependent upon the status of the previous annotations a series of functional impact predictions are performed. For nonsynonymous variants, the predicted impact of the amino acid substitution on protein function is determined based upon the SIFT (Ng and Henikoff 2001), Polyphen-2 (Adzhubei et al 2010), and Condel algorithms (González-Pérez and López-Bigas 2011). For all coding variants, including in-frame insertions and deletions, a Log Ratio *E-value* score of variants, which is the log ratio of the E-value of the HMMER (Eddy 2009) match of PFAM protein motifs (Finn et al 2008) between the variant and original amino acid sequences (Clifford et al 2004). This score has been shown to be capable of accurately identifying known deleterious mutations. A suggested threshold is scores with a LogR.*E-value* greater than 0.7 for predicted damaging variants. More importantly, this score measures the fit of a full protein sequence to a PFAM motif, therefore multinucleotide substitutions or separate substitutions on the same haplotype are capable of being scored by this approach. As phased genomes gain in prevalence, phased nonsynonymous variants can be analyzed for their combined impact on protein function. For truncating variants (nonsense and frameshift) the percentage of the conserved upstream and downstream coding sequence removed by the truncation (conserved elements), taking into account alternate start sites, is determined and utilized to predict whether the truncation is damaging or not. The threshold for prediction of a damaging truncation removal of >4% of the conserved portion of the protein, a threshold with the greatest accuracy as defined empirically in (Hu and Ng 2012).

**Splicing Impact, Inferred and Predicted**

Variants falling near exon-intron boundaries are evaluated for their impact on splicing in a couple of ways. One method is a simple determination of whether or not the variant impacts the invariant splice donor and acceptor sequences – returning an annotation that a splice donor or acceptor is damaged. A second method is a prediction of the impact of variants nearby a gene splice junctions based on the maximum entropy method of maxENTscan (Yeo and Burge 2004). Maximum entropy scores are calculated for the original and variant sequence independently, and considered for their impact on splicing. Changes from a positive original score to a negative variant score suggest a splice site disruption. Variants falling within exons are also analyzed for their impact on exonic splicing enhancers and/or silencers (ESE / ESS). The number of ESE and ESS sequences created or destroyed is based on the hexanucleotides reported as potential exonic splicing regulatory elements in (Stadler et al 2006) and shown to be the most informative for identification of splice-affecting variants by (Woolfe et al 2010).

**Regional Information**

Regional information refers to sequence-based, cross-species inferred and structural characteristics of the specific region of the genome containing the genetic variant. Two primary annotations are the repeat structure of the genomic region and its conservation across species. Segmental duplications, duplicated regions of the genome which increase the likelihood of mismapped reads and false variant calls, are annotated. All variants are also associated with conservation information in two ways. First, variants are associated with conserved elements from the phastCons conserved elements at various depths of conservation (Siepel 2005). These conserved elements represent potential functional elements preserved across species. Conservation is also assessed at the specific nucleotide positions impacted by the variant by the phyloP method (Pollard 2010).

**Population-Based Information**

Population-based information refers to known rates and identifiers in populations already sequenced or genotyped. These variants are generally associated with dbSNP identifiers (Sherry et al 1999), however the SG-ADVISER platform also dynamically tracks and updates the allele frequency of all variants processed through the system and derived from reference panels. SG-ADVISER reports the population allele frequencies for all HapMap populations (International HapMap Consortium et al 2003) as well as allele frequencies in available reference populations including the 1,000 genomes project (1000 Genomes Project Consortium 2010), the NHLBI exomes project, and Scripps Translational Science Institute Wellderly samples. The Wellderly are a collection of individuals over the age of 80 with no signs of common chronic diseases.

**Regulatory Variants**

All variants, regardless of their genomic position, are associated with predicted transcription factor binding sites (TFBS) and scored for their potential impact on transcription factor binding. Predicted TFBS are pre-computed by utilizing the human transcription factors listed in the JASPAR and TRANSFAC transcription-factor binding profile to scan the human genome using the MOODS algorithm (Wasserman and Sandelin 2004, Wingender 1996, Korhonen 2009). The probability that a site corresponds to a TFBS is calculated by MOODS based on the background distribution of nucleotides in the human genome. TFBS are called at a relaxed threshold within (p-value < 1∙10^-6^) in conserved, hypersensitive, or promoter regions, and at a more stringent threshold (p-value < 1∙10^-8^) for all other locations in order to capture sites that are more likely to correspond to true functional TFBS. Conserved and hypersensitive sites correspond to the phastCons conserved elements, Encode DNASE hypersensitive sites annotated in UCSC genome browser, while promoters corresponds to 2kb upstream of known gene transcription start sites, promoter regions annotated by TRANSPro, and transcription start sites identified by SwitchGear Genomics ENCODE tracks. The potential impact of variants on TFBS are scored by calculating the difference between the variant and original sequence scores using the position weighted matrix method described in Stormo 2000 and shown to identify regulatory variants in Andersen et al 2008. A suggested threshold for damaged TFBS is either deleted TFBS or those with a delta score of less than -7.0. Variants known to influence expression levels, as determined by eQTL analyses are also annotated from the NCBI GTEx database (Baker 2012).

Variants falling within 3’Untranslated Regions (3’UTRs) are analyzed for their impact on microRNA binding in two different ways. First, all 3’UTRs are associated with pre-computed microRNA binding sites using the TargetScan algorithm and database (Lewis et al 2005). Variant 3’UTR sequences are rescanned by TargetScan in order to determine if microRNA binding sites are lost due to the impact of the variation. Directly impacted microRNA binding sites are listed as well, and the binding strength of the microRNA with its original and variant binding site is calculated by the RNAcofold algorithm to return a ∆∆G score for the change in microRNA binding strength induced by introduction of the variant (Hofacker et al 2003). For microRNA transcripts (rather than their binding sites) bearing variants, a change in folding and binding energy, based on annealing with the consensus binding site, is also calculated by the RNAcofold algorithm. Moreover, a list of predicted lost and gained targets due to the new microRNA sequence is determined using the TargetScan algorithm to scan the novel microRNA sequence against known transcript 3’UTRs.

**Clinical Annotations**

Clinical annotations include both return of information contained within clinical variant databases as well as predicted clinical influences based upon the synthesis of gene-phenotype relationships and gene-variant impact predictions (described in *Synthetic Annotations*). SG-ADVISER, on a variant by variant basis determines whether the specific reported variant is contained with the Human Gene Mutation Database (HGMD) (Krawczak et al 2000), PharmGKB (Thorn et al 2010), GET-Evidence (Ball et al 2012), and the COSMIC Database (Bamford et al 2004). HGMD cross-reference returns the disease associated with the genetic variant (HGMD subscription information is required for retrieval of primary HGMD data), PharmGKB cross-reference returns the PharmGKB entry name and the drug whose metabolism is perturbed by the variant, GET-Evidence cross-reference returns the inheritance, penetrance, severity, and treatability of the variant and disease if it is known, and COSMIC Database cross reference returns the number of cancer samples in the COSMIC database bearing that specific variant. If the nearest gene, rather than the specific variant, is a gene known to be clinically relevant, it’s association with disease as annotated by either OMIM (McKusick 1998), HGMD (Krawczak et al 2000), the or NCBI Genetic Association Database (Becker et al 2004) is returned. Finally, if the variant falls nearest to a gene associated with cancer, that information is returned via cross-reference to the COSMIC database (Bamford et al 2004), Memorial Sloan Kettering Cancer Center (Higgins et al 2007), Atlas Oncology (http://atlasgeneticsoncology.org), the Sanger Cancer Gene Census (Futreal et al 2004), or network residence nearby known cancer genes. Drugs known to target the gene are also returned from DrugBank (Wishart et al 2006).

Finally, two different modified American College of Medical Genetics (ACMG) scores are returned, one based upon variants, or variants in genes known to be causally associated with a phenotype (ACMG Clinical) and a second score which includes genes known to carry genetic variants that are statistically associated risk factors for the development of a disease (Research). The ACMG scoring guidelines (Richards et al 2007) with categories 1-6 are modified to include a 2* and 4* category to provide more granularity to variant stratification. However, variants of category 1-2* are of most clinical relevance and category 6 contains more common risk factors for disease. ACMG category inclusion criteria are defined at (<http://genomics.scripps.edu/ADVISER/ACMG.jsp>).

**Gene Networks, Pathways, Biological Process and Molecular Functions**

This category of annotation includes information that can link genes and variants to one another based upon biological, molecular, and/or functional relationships. These relationships are useful for pathway or process based collapsed association methods or inferring the phenotypic influence of particular variants. In our specific implementation Reactome pathways (Joshi-Tope et al 2005) and gene ontology biological processes (Ashburner et al 2000) of the nearest gene are utilized to provide biological relationships. Disease Ontology (Osborne et al 2009) annotations are utilized to provide phenotypic relationships. Protein domain information and molecular functions (as annotated by Gene Ontology) utilized by the nearest gene are used to provide molecular and functional relationships.

**Computational Process**

The computational processes underlying SG-ADVISER output do not necessarily follow the structure given by *Annotation Classes*. Rather, annotation execution proceeds in highly parallel fashion on a high-performance computational cluster and includes classes of variant annotations that are entirely independent of one another, serially dependent annotations whose execution are dependent upon the completion and status of prior annotations, and synthetic annotations that generate new information through the combination of multiple annotation outputs.

**Data Input**

The input requirements are solely a list of variants including the chromosome, start position, end position, and reference and variant alleles – transferred via a network connection for automated processing or provided via a local file. One of a few standard file formats, including VCF (variant call format), Complete Genomics native file format, or basic tab delimited BED-like file format are accepted, but then converted to the following tab-delimited structure: 1.) Haplotype number (can be a placeholder); 2.) Chromosome (with syntax: “chr1”, “chr22”, “chrX”, etc.); 3.) Start position (0-based coordinates); 4.) End position (0-based coordinates); 5.) Variant type (“snp”, “ins”, “del”, “delins”); 6.) Reference allele(s); 7.) Observed allele(s); and 8.) Notes.

**Pre-Annotated Database**

Annotations for all observed variants, including all variants reported in dbSNP, or other databases mentioned above, as well as any annotations completed by SG-ADVISER on novel variants, are stored within a pre-annotation database. Annotations are stored in a scalable database capable of quick queries based upon physical location alone – in our case a non-relational database such as MongoDB. Previously annotated variants are extracted from this database, and novel variant annotations are stored within the database upon completion in order to speed up the annotation of all subsequent genomes. Annotations for variants not found in the database are computed *de novo* in an automated parallel computing environment as described below. Subsequent retrieval of pre-annotated variants contained within variant files submitted for annotation is based purely upon physical coordinate based queries against the pre-annotation database.

**Transcript-Based Annotations**

Annotations of this category rely upon the mapping of variants relative to known gene transcripts. This includes, for example, the nearest gene or transcript, the position of a variant in the genome relative to a transcript, the position of the variant within the transcript (e.g. exonic, intronic, upstream, etc.) and the position of the variant relative to functional elements or “gene components” of the transcript (e.g. untranslated regions, splice junctions, distance from coding start and stop sites etc.). Annotation depends upon a database of the physical location of all genes and gene components and a measurement of the physical distance or occupancy of a variant relative to these gene components. In more complex instances, a reference genome sequence is utilized to extract the genomic sequence relevant to the annotation in question; the sequence is processed based upon the reported coding frame and then trimmed based upon proximity to gene components or converted to other biological sequences by utilizing the standard genetic code to produce input formats compatible with downstream tools. Based upon this information a series of transcript based annotations are produced. The simplest case is annotations dependent solely upon the identity of the nearest gene. Prior knowledge regarding the nearest gene/transcript is produced from knowledgebases, including the type of gene, the relationship of the gene to phenotypes and biological processes as determined by clinical phenotype database (OMIM, HGMD, COSMIC, Disease Ontology, other cancer gene databases etc.), relationship of the gene to other compounds (e.g. DrugBank), and relationship of the nearest gene to biological pathways, networks, or molecular function (e.g. Gene Ontology, Reactome Pathways, etc.). These cross-references are based upon gene synonym tables, for example those provided by the UCSC Genome Browser. More complex cases require utilization of the position of the variant relative to the transcript body as well as the sequence of the surrounding nucleotide context. In these cases, given the position of the variant within the transcript body, a series of annotations and predictions that are dependent upon the location of a variant relative to functional transcript elements and calculation of results based upon the specific surrounding nucleotide sequence are produced - these include inferred and predicted influences upon splicing, and splicing machinery, as well as inferred and predicted influences upon microRNA binding sites within the untranslated regions. Finally, given the gene type and position within the transcript, a series of annotations are produced that depend upon defining the protein produced by the gene and the changes to the amino acid sequence based upon the standard genetic code. These annotations include the position of the variant within the protein sequence and the distance of the variant from the coding start and stop sites, the affected amino acids, and predictions which utilize the protein sequence and perturbed amino acids including functional predictions (SIFT, Polyphen-2, Condel), and protein domain matching and scoring (e.g. HMMER scanning of the protein sequence against protein family models).

**Functional Element-Based Annotations**

This class of annotation does not depend upon the identity of the nearest gene, but rather the characteristics of the genomic position itself. The reference components of these annotations could sometimes be considered synthetic annotations (see *Synthetic Annotations*) on their own; however the considered synthetic elements are based upon synthesis of data prior to any variant annotation in order to identify functional elements of interest in the genome (see *Synthetic Annotations* below). For example, transcription factor binding site motifs are scanned against the genome, their scores and positions relative to known genomic elements are determined, and specific transcription factor binding sites are called based upon the synthesis of this information. In the annotation process these elements are predefined and variants are mapped directly to these elements and their functional impact, if any, are predicted based upon the sequence context. A basic functional element-based annotation is based purely upon the residence of a variant within a genomic region with a particular characteristic - for example regions denoted as Segmental Duplication. Databases of genomic regions, such as Segmental Duplications, are maintained and variants are mapped against these bins to determine whether they land within the span of the element. Other examples including conserved elements or base-specific conservation levels. More complex annotations rely upon the residence of a variant within a functional bin along with calculations that depend upon the identity of the original and variant sequence. These annotations including impact scoring for variants within transcription factor binding sites, or determination of the influence of variants upon microRNA genes. Thus, for functional element-based annotations, the locations of functional elements in the genome are either defined *a priori* in knowledgebases and mapped to the reference genome based upon physical location, or defined by searching for patterns within the reference genome that define functional elements. The bins defined by physical location coordinates are utilized to determine the residence of genetic variants within a functional element and the sequence of those bins is extracted from the reference genome, modified with the variant information, and scored using the various predictive algorithms described above.

**Variant-Based Annotations**

This class of annotation depends solely upon prior or generated knowledge about the variant itself. For example, numerous sequencing and genotyping projects have catalogued the type and frequency of particular variants in different reference populations. These population based annotations are either generated from reference data or drawn from knowledgebase and reported – for example as known identifiers (dbSNP ID’s), frequency in different reference populations, or frequency in different samples of known disease-status (e.g. tumor genomes). Most clinical annotations, and all other prior knowledge derived from variant-centric databases – for example the reported associations of variants with particular molecular or physiological phenotypes in HGMD , GET-Evidence, or eQTL databases are variant-based. All cross-referencing is done based upon conversion to data within external knowledgebases to physical coordinates in the genome (as defined in *Data Inputs*), execution of the SG-ADVISER annotation pipeline, and deposition of the resulting annotations in our pre-annotated database.

**Synthetic Annotations**

Finally, certain annotations depend upon the synthesis of multiple annotations. These annotations are split into two types, synthetic annotations generated prior to use in the SG-ADVISER pipeline and utilized as knowledgebases for other annotations, or synthetic annotations that depend upon the output of active variant annotation. Pure synthesis of prior data to define functional elements is exemplified by the definition of transcription factor binding sites to be used to produce, for example, functional element based annotations as described above. Hybrid-synthetic annotations involve the combination and merging of any subset of the previously described annotations in order to produce a novel layer of annotation. An example is the prediction of impact of truncating variants – which relies upon the definition of a truncating variant, its position within the coding sequence, and the amount of flanking conserved sequence removed by the variant. In this case, production of a synthetic annotation requires information from the annotation process as well as reference to the various knowledgebases maintained by SG-ADVISER. Finally, there exist annotations that are pure synthesis of variant based annotations completed in prior steps. An example of this sort of annotation is ACMG scoring, which relies upon the identity of the nearest gene, the association of the nearest gene with disease phenotypes, and the predicted impact of the variant upon the gene based upon the above described coding, splicing, and regulatory prediction tools. These annotations rely upon the combination of previous annotation outputs through logical operators and rules to define a novel annotation result.

**User Interface**

**Summary:**

SG-ADVISER UI is a visualization tool that allows scientists of with little to no computational expertise to easily and quickly view, manipulate, sort, filter and extract fragments of the SG-ADVISER output file. A variety of custom and advance filters allow filtering based on any combination of sample and annotation information. The intent is to enable all common and/or suggested variant sorting and filtering strategies in a manner accessible to a broad group of investigators looking to use genome sequencing in research.

**Availability and Implementation**: SG-ADVISER UI is written in Java and is freely available for download along with a User Guide at <http://genomics.scripps.edu/ADVISER/>

Note, Windows users must be sure to start the SG-ADVISER UI using the Windows Batch File – the software will start using the Executable Jar File but will not work properly.

**File formats:**

The tool accommodates the SG-ADVISER output format. See <http://genomics.scripps.edu/ADVISER/Result_Desc.jsp> for a description of the output format. Genotype information can be loaded from the original variant file in VCF or masterVar format.

**Requirement and Performance:**

Basic SG-ADVISER UI functionality requires Java JRE 1.5 or higher, but Java JDK 1.6 is recommended (JDK is the full Java Development Kit). The software is known to work with Windows XP and Windows 7, Mac OS X, and Linux. SG-ADVISER UI is platform independent: any OS with Java should be able to run the program.

The tool runs best with a current 64-bit processor, and a 64-bit Java version. The amount of RAM required depends on the size of the file you wish to view. A machine with 2 GB RAM will allow 1GB input file; 8GB RAM will allow 4GB file, and so forth. A typical output file derived from a trio undergoing exome sequencing is well under 0.5 GB. Annotations for a typical trio load in under 5 seconds.

**Graphical display:**

Variant data is displayed in the UI as a single table where each row corresponds to a single variant and the columns are the output from SG-ADVISER. Due to the large number of variants per genome, and the resultant large annotation file size, the data is presented in paginated fashion, with each page containing 500 variants. Users may manually navigate through pages using the “Next/Previous Page” buttons at the bottom right corner of the UI. To accelerate loading time, a multithreaded process is initiated allowing nearly immediate viewing of the leading pages of variants while the later pages continue to load into memory. A progress bar indicates the status and completion of file loading. Upon completion, sorting and filtering functions are enabled.

**User Interface Functions**

Once an annotation file is loaded, other functionalities become available.

**File → Load & Compare VCF / Complete Genomics File:**

In order to respect the privacy of our users, a SG-ADVISER annotation run does not automatically import genotype information. Genotype information can be loaded into the UI locally by first downloading and loading in the resultant annotation file and then loading the original variant file. Imported genotypes will populate the first column (‘Imported_Genotypes’) of the SG-ADVISER UI. We do not maintain user genotype information on the SG-ADVISER system. The Load and Compare function will accept any variant file and import in genotypes for filtering. Genotypes for a typical trio load in under 5 seconds.

**Sort → Sort**

File sorting proceeds through a heap sort alphanumeric algorithm that enables viewing of the top sorted variants while the lower ranked variants continue to be sorted in the background. **Warning**: sorting an unfiltered variant file can be very time consuming – filtering should be done prior to sorting. The currently viewable page can also be sorted by clicking on the header of the column to be sorted.

**Filtering**

1. Simple Filter: String-matched based filtration on a single column directly in the UI interface.
2. Advanced Filter: The advanced filter button allows filtering using three annotation columns and matched terms.
3. Custom Filters: The custom filters dropdown contains common and suggested filtering strategies.

**Simple Filter:** simple filtering can simply be accomplished by selecting the column for which one wants to filter, entering the filter criteria, and a simple string-match based filtration will be executed.

**Advanced Filter**: advanced filtering can be accomplished by pressing the “Advanced Filter” button, entering in the filtration criteria by selecting columns to filter on in the drop down boxes, text for the string match in the text-box, and a logical operator to join up to three different filtration criteria. Note, this functionality is most useful when attempting to filter on multiple columns using an “OR” logical operator. “AND” filters can be simply executed by performing multiple simple filters in series.

**Undo/Redo**: at any time, the user may step forward and/or backward through the different filters applied by pressing the forward or back arrows.

**Custom Filters:** Custom filters are pre-designed filters useful for most common filtration tasks. They are defined below:

Custom Filters Description

| Filter Name | Description |
| --- | --- |
| Coding Variants | All variants impacting the protein coding sequence of a gene: i.e. all possible coding impacts except synonymous variants. |
| Coding & Splice Variants | Coding Variants plus variants annotated to damage splice donor and acceptor sites. |
| Coding and Splice Variants with Frequency | Coding and Splice Variants plus a user defined frequency threshold in as observed in the 1000 Genomes, 69 publically available Complete Genomics genomes and the Scripps Wellderly population. |
| Known Disease | '1' values of the ACMG-Score columns |
| Clinical Known and Predicted Causing Variants | All entries in the column 'ACMG Score Clinical/Disease Entry/Explanation' receiving a modified ACMG categorization of 1, 2, or 2*. See <http://genomics.scripps.edu/ADVISER/ACMG.jsp> for ACMG scoring criteria. |
| Research Known and Predicted Causing Variants | All entries in the column 'ACMG Score Research/Disease Entry/Explanation' receiving a modified ACMG categorization of 1, 2 and 2*. See <http://genomics.scripps.edu/ADVISER/ACMG.jsp> for ACMG scoring criteria. |
| Cancer Genes | All genes annotated as cancer genes by the Sanger Cancer Gene Census, Memorial Sloan Kettering Cancer Center or Atlas Oncology. |
| Pharmacogenetic | All variants curated by PharmGKB. Currently inactive. |
| Truncating Variants | All Frameshift and Nonsense variants. |
| Novel Variants | Potential novel variants – i.e. variants not catalogued in dbSNP. |
| Chromosome Position | All variants within a user defined region. |
| Existing Valid Values | Filter any column for any value not including the “-“ character representing no data. |
| IDIOM Filter | A (currently) simple trio filter which allows for the selection of an affected proband and two unaffected parents for the execution of a combination of filters including: 1) an inheritance based filter to select only variants inherited in a de-novo dominant, recessive, or compound heterozygous fashion, 2) selection of only variants that pass the “Coding and splice Variants with Frequency” filter with a 1% allele frequency cutoff, and 3) the option to include a variant quality filter. |

**Other functionality:**

**Resizing and Rearrangement** – Columns are free to be re-sized or rearranged allowing the user to optimize viewing of columns of interest.

**Commenting** – the last column in the SG-ADVISER comment can be used to input user comments.

**Save** – Post-filtered annotation data can be saved to file for further manipulation in 3^rd^ party programs. The saved file will include any comments input by the user. The saved file can also be re-loaded later for further manipulation.

**Statistics** – calculation of statistics of variants passing filter criteria. The following describes the inclusion requirements for each variant under every category generated by the Statistics tool of the SG-ADVISER User Interface.

**Coding SNPs** – all SNPs where Coding_Impact is Nonsense, Nonsynonymous or Synonymous;

**Nonsynonymous SNPs –** all SNPs where Coding Impact is Nonsynonymous;

**Synonymous SNPs -** all SNPs where Coding Impact is Synonymous;

**Nonsense SNPS -** all SNPs where Coding Impact is Nonsense;

**Untranslated Region SNPs -** all SNPS where Location is 5UTR or 3UTR;

**Intronic SNPs -** all SNPs where Location contains Intron

**Total SNPs –** total SNPs present in file

**Coding Insertions –** all insertions where Coding_Impact contains Frameshift, InterCodon_In_Frame_Insertion or In_Frame_Insertion;

**In-frame Insertions** – In_Frame_Insertions in Coding_Impact;

**Out-of-frame Insertions -** InterCodon_In_Frame_Insertion in Coding_Impact;

**Frameshift Insertions –** Frameshift in Coding_Impact

**Untranslated region Insertions –** 5UTR or3UTR in Location

**Intronic Insertions –** Intron in Location

**Total Insertions –** total number of insertions in file(array)

**Coding Deletions** – all deletions where Coding_Impact contains Frameshift, In_Frame_Deletions_One_Altered_Codon, In_Frame_Deletion

**In-frame Deletions –** In_Frame_Deletions at Coding_Impact

**Inter-Codon Deletions –** In_Frame_Deletion_One_Altered_Codon at Coding_Impact

**Frameshift Deletions –** Frameshift deletions at Coding Impact

**Untranslated region Deletions –** 5UTR or 3UTR at Location

**Intronic Deletions –** Intron at Location

**Total Deletions** – total number of deletions if file

**Coding rearrangements** – indels where Coding Impact column contains either Frameshift or In_Frame_Rearrangement

**In-frame rearrangements –** In_Frame rearrangement in Coding_Impact

**Frameshift rearrangements –** indels where 5UTR or 3UTR is present in column Location

**Untranslated region rearrangements -** 5UTR or 3UTR at Location

**Intronic rearrangements -** Intron at Location

**Total rearrangements –** total number of indels

**Total number of variants**

**Conserved Element (snp, ins, del or delins) - *ConservedXX*** - Conserved elements and nucleotide specific conservation levels. "XX" in column header (i.e. 46way) corresponds to the species considered for conservation analysis. Conserved elements are determined by PhastCons (Siepel et al. 2005). Nucleotide specific conservation level is determined by PhyloP (Pollard et al. 2010).

**TFBS (snp, ins, del or delins) –** Predicted transcription factor binding sites impacted by the variant. Number of elements that have values in the TFBS_deltaS column that are located upstream, is a conserved element and its value is smaller than “-7” or are deleted;

**Rate** - is the division of TFBS (snp, ins, del or rear) by the total number of elements that have a value in the TFBS_DeltaS column.

pre-miRNA Disrupting (SNPS, Insertions, Deletions, Rearrangements)

**ESE-BS deletion (snp, del, ins or delins)** – variants that contain “DELETED” values in the ESE_sites column;

**Rate** – number of ESE-BS deletions divided by the total number of variants that have “DELETED” or “CREATED ” values in the ESE _sites column;

**ESS-BS induction (snp, del, ins or delins)** – variants that contain “CREATED” values in the ESS_sites column;

**Rate** – number of ESS-BS deletions divided by the total number of variants that have “DELETED” or “CREATED ” values in the ESE _sites column;

**ESS-BS deletion (snp, del, ins or delins)** – variants that contain “DELETED” values in the ESS_sites column;

**Rate** – number of ESS-BS deletions divided by the total number of variants that have “DELETED” or “CREATED ” values in the ESS _sites column;

**ESS-BS induction (snp, del, ins or delins)** – variants that contain “CREATED” values in the ESS_sites column;

**Rate** – number of ESS-BS induction divided by the total number of variants that have “DELETED” or “CREATED ” values in the ESS _sites column;

**miRNA-BS disrupting (snp, del, ins or delins)** – variants that contain “DELETED” values in the miRNA_BS_impact column;

**Rate** – number of miRNA-BS disturbing divided by the total number of variants that have “DELETED” or “CREATED ” values in the miRNA-BS column;

**Splicing Change(snp, del, ins, delins)** - the entries in the "Splicing_Prediction(MaxENT)" columns that have Splicing_Change value;

**Rate Splicing Change(snp, del, ins, delins) - t**he entries in the "Splicing_Prediction(MaxENT)" column that have Splicing_Change value divided by the total number of the entries in the "Splicing_Prediction(MaxENT)" column that have any value;

**Protein Motif disrupting (snp, del, ins, delins) –** variants that have values of logRE > 0.7 in the Protein_Domains_Impact(LogRE) column;

**Rate** - Protein Motif disrupting (snp, del, ins, delins) divided by the total number of variants in the Protein_Domains_Impact(LogRE) column that have values logRE > 0.0

**Nonsense SNPs -** all SNPs where Coding Impact is Nonsense;

**Rate** - Nonsense SNPs/Coding SNPs

**Frameshift Structural Variants** - fshift_del + fshift_ins + fshift_rear;

**Rate** – (fshift_del + fshift_ins + fshift_rear)/ coding_ins + coding_del + coding_rear;

**Splicing Change Variants** – all entries in the Splicing_Prediction(MaxENT) column that have “Splicing Change” values as entries;

**Rate –** splice_change/splice_site (all entries that have either “Splicing_change”, “No_splicing_change”, “Unconventional_Splice_Site” as entries in the column "Splicing_Prediction(MaxENT)";

**Probably Damaging nscSNPs** – variants that have “probably damaging” values in the Protein_Impact_Prediction(Polyphen) column;

**Probably Damaging Rate =** Probably Damaging nscSNPs/ Nonsynonymous variants;

**Possibly Damaging nscSNPs** - variants that have “possibly damaging” values in the Protein_Impact_Prediction(Polyphen) column;

**Rate** = Possibly Damaging nscSNPs / Nonsynonymous variants;

**Protein motif damaging Variants** – variants that have values of logRE > 0.7 in the Protein_Domains_Impact(LogRE) column;

**Rate** - variants that have values of logRE > 0.7 in the Protein_Domains_Impact(LogRE) column divided by the total number of variants that have logRE > 0.0 in the Protein_Domains_Impact(LogRE) column;

**TFBS Disrupting Variants** – the number of variants that have in the TFBS_deltsS column values “DELETED” or values that are smaller than “-7”;

**Rate** = TFBS Disrupting Variants divided by the number of variants that have values < 0 (or “DELETED”) in the “TFBS_deltaS” column;

**pre-miRNA Disrupting Variants** – number of variants that have values > 0 or “DELETED” in the miRNA_folding_deltaG column;

**Rate** – number of variants that have values > 0 or “DELETED” in the miRNA_folding_deltaG column divided by the total number of variants;

**miRNA-BS Disrupting Variants** – all variants that contain “DELETED” values in the miRNA_BS_impact column;

**Rate** - all variants that contain “DELETED” values in the miRNA_BS_impact column divided by the total number of variants that have values in the miRNA_BS_impact column;

**Total Likely Functional Variants –** total number of variants that are: nonsense + Polyphen probably damaging + Polyphen possibly damaging

TFBS Disrupting Variants + ESE-BS deletion Variants + ESE-BS induction Variants +ESS-BS deletion Variants + ESS-BS induction Variants + pre-miRNA Disrupting Variants + Splicing Changed variants + Protein Motif disrupting variants;

**Rate -** total likely functional variants divided by the number of lines;

**References**

1000 Genomes Project Consortium. A map of human genome variation from population-scale sequencing. Nature. 2010 Oct 28;467(7319):1061-73.

Adzhubei IA, Schmidt S, Peshkin L, Ramensky VE, Gerasimova A, Bork P, Kondrashov AS, Sunyaev SR. A method and server for predicting damaging missense mutations. Nat Methods. 2010 Apr;7(4):248-9.

Andersen MC, Engström PG, Lithwick S, Arenillas D, Eriksson P, Lenhard B, Wasserman WW, Odeberg J. In silico detection of sequence variations modifying transcriptional regulation. PLoS Comput Biol. 2008 Jan;4(1):e5.

Ashburner M, Ball CA, Blake JA, Botstein D, Butler H, Cherry JM, Davis AP, Dolinski K, Dwight SS, Eppig JT, Harris MA, Hill DP, Issel-Tarver L, Kasarskis A, Lewis S, Matese JC, Richardson JE, Ringwald M, Rubin GM, Sherlock G. Gene ontology: tool for the unification of biology. The Gene Ontology Consortium. Nat Genet. 2000 May;25(1):25-9.

Baker M. Biorepositories: Building better biobanks. Nature. 2012 Jun 6;486(7401):141-6. doi: 10.1038/486141a.

Ball MP, Thakuria JV, Zaranek AW, Clegg T, Rosenbaum AM, Wu X, Angrist M, Bhak J, Bobe J, Callow MJ, Cano C, Chou MF, Chung WK, Douglas SM, Estep PW, Gore A, Hulick P, Labarga A, Lee JH, Lunshof JE, Kim BC, Kim JI, Li Z, Murray MF, Nilsen GB, Peters BA, Raman AM, Rienhoff HY, Robasky K, Wheeler MT, Vandewege W, Vorhaus DB, Yang JL, Yang L, Aach J, Ashley EA, Drmanac R, Kim SJ, Li JB, Peshkin L, Seidman CE, Seo JS, Zhang K, Rehm HL, Church GM. A public resource facilitating clinical use of genomes. Proc Natl Acad Sci U S A. 2012 Jul 24;109(30):11920-7.

Bamford S, Dawson E, Forbes S, Clements J, Pettett R, Dogan A, Flanagan A, Teague J, Futreal PA, Stratton MR, Wooster R. The COSMIC (Catalogue of Somatic Mutations in Cancer) database and website. Br J Cancer. 2004 Jul 19;91(2):355-8.

Becker KG, Barnes KC, Bright TJ, Wang SA. The genetic association database. Nat Genet. 2004 May;36(5):431-2.

Clifford RJ, Edmonson MN, Nguyen C, Buetow KH. Large-scale analysis of non-synonymous coding region single nucleotide polymorphisms. Bioinformatics. 2004 May 1;20(7):1006-14.

Eddy SR. A new generation of homology search tools based on probabilistic inference. Genome Inform. 2009 Oct;23(1):205-11.

Finn RD, Tate J, Mistry J, Coggill PC, Sammut SJ, Hotz HR, Ceric G, Forslund K, Eddy SR, Sonnhammer EL, Bateman A. The Pfam protein families database. Nucleic Acids Res. 2008 Jan;36(Database issue):D281-8.

Fujita PA, Rhead B, Zweig AS, Hinrichs AS, Karolchik D, Cline MS, Goldman M, Barber GP, Clawson H, Coelho A, Diekhans M, Dreszer TR, Giardine BM, Harte RA, Hillman-Jackson J, Hsu F, Kirkup V, Kuhn RM, Learned K, Li CH, Meyer LR, Pohl A, Raney BJ, Rosenbloom KR, Smith KE, Haussler D, Kent WJ. The UCSC Genome Browser database: update 2011. Nucleic Acids Res. 2011 Jan;39(Database issue):D876-82.

Futreal PA, Coin L, Marshall M, Down T, Hubbard T, Wooster R, Rahman N, Stratton MR. A census of human cancer genes. Nat Rev Cancer. 2004 Mar;4(3):177-83.

González-Pérez A, López-Bigas N. Improving the assessment of the outcome of nonsynonymous SNVs with a consensus deleteriousness score, Condel. Am J Hum Genet. 2011 Apr 8;88(4):440-9.

Higgins ME, Claremont M, Major JE, Sander C, Lash AE. CancerGenes: a gene selection resource for cancer genome projects. Nucleic Acids Res. 2007 Jan;35(Database issue):D721-6.

Hofacker IL. Vienna RNA secondary structure server. Nucleic Acids Res. 2003 Jul 1;31(13):3429-31.

Hu J, Ng PC. Predicting the effects of frameshifting indels. Genome Biol. 2012 Feb 9;13(2):R9.

International HapMap Consortium. The International HapMap Project. Nature. 2003 Dec 18;426(6968):789-96.

Joshi-Tope G, Gillespie M, Vastrik I, D'Eustachio P, Schmidt E, de Bono B, Jassal B, Gopinath GR, Wu GR, Matthews L, Lewis S, Birney E, Stein L. Reactome: a knowledgebase of biological pathways. Nucleic Acids Res. 2005 Jan 1;33(Database issue):D428-32.

Korhonen J, Martinmäki P, Pizzi C, Rastas P, Ukkonen E. MOODS: fast search for position weight matrix matches in DNA sequences. Bioinformatics. 2009 Dec 1;25(23):3181-2.

Krawczak M, Ball EV, Fenton I, Stenson PD, Abeysinghe S, Thomas N, Cooper DN. Human gene mutation database-a biomedical information and research resource. Hum Mutat. 2000;15(1):45-51.

Lewis BP, Burge CB, Bartel DP. Conserved seed pairing, often flanked by adenosines, indicates that thousands of human genes are microRNA targets. Cell. 2005 Jan 14;120(1):15-20.

McKusick VA. Mendelian Inheritance in Man. A Catalog of Human Genes and Genetic Disorders. Baltimore: Johns Hopkins University Press, 1998 (12th edition).

Ng PC, Henikoff S. Predicting deleterious amino acid substitutions. Genome Res. 2001 May;11(5):863-74.

Osborne JD, Flatow J, Holko M, Lin SM, Kibbe WA, Zhu LJ, Danila MI, Feng G, Chisholm RL. Annotating the human genome with Disease Ontology. BMC Genomics. 2009 Jul 7;10 Suppl 1:S6.

Pollard KS, Hubisz MJ, Rosenbloom KR, Siepel A. Detection of nonneutral substitution rates on mammalian phylogenies. Genome Res. 2010 Jan;20(1):110-21.

Richards CS, Bale S, Bellissimo DB, Das S, Grody WW, Hegde MR, Lyon E, Ward BE; Molecular Subcommittee of the ACMG Laboratory Quality Assurance Committee. ACMG recommendations for standards for interpretation and reporting of sequence variations: Revisions 2007. Genet Med. 2008 Apr;10(4):294-300.

Thorn CF, Klein TE, Altman RB. Pharmacogenomics and bioinformatics: PharmGKB. Pharmacogenomics. 2010 Apr;11(4):501-5.

Sherry ST, Ward M, Sirotkin K. dbSNP-database for single nucleotide polymorphisms and other classes of minor genetic variation. Genome Res. 1999 Aug;9(8):677-9.

Siepel A, Bejerano G, Pedersen JS, Hinrichs AS, Hou M, Rosenbloom K, Clawson H, Spieth J, Hillier LW, Richards S, Weinstock GM, Wilson RK, Gibbs RA, Kent WJ, Miller W, Haussler D. Evolutionarily conserved elements in vertebrate, insect, worm, and yeast genomes. Genome Res. 2005 Aug;15(8):1034-50.

Stadler MB, Shomron N, Yeo GW, Schneider A, Xiao X, Burge CB. Inference of splicing regulatory activities by sequence neighborhood analysis. PLoS Genet. 2006 Nov 24;2(11):e191.

Stormo GD. DNA binding sites: representation and discovery. Bioinformatics. 2000 Jan;16(1):16-23.

Wasserman WW, Sandelin A. Applied bioinformatics for the identification of regulatory elements. Nat Rev Genet. 2004 Apr;5(4):276-87.

Wingender E, Dietze P, Karas H, Knüppel R. TRANSFAC: a database on transcription factors and their DNA binding sites. Nucleic Acids Res. 1996 Jan 1;24(1):238-41.

Wishart DS, Knox C, Guo AC, Shrivastava S, Hassanali M, Stothard P, Chang Z, Woolsey J. DrugBank: a comprehensive resource for in silico drug discovery and exploration. Nucleic Acids Res. 2006 Jan 1;34(Database issue):D668-72.

Woolfe A, Mullikin JC, Elnitski L. Genomic features defining exonic variants that modulate splicing. Genome Biol. 2010;11(2):R20.

Yeo G, Burge CB. Maximum entropy modeling of short sequence motifs with applications to RNA splicing signals. J Comput Biol. 2004;11(2-3):377-94.
